# Transformer-Based Soft Actor–Critic for UAV Path Planning in Precision Agriculture IoT Networks

**DOI:** 10.3390/s25247463

**Published:** 2025-12-08

**Authors:** Guanting Ge, Mingde Sun, Yiyuan Xue, Svitlana Pavlova

**Affiliations:** College of Software, Shanxi Agricultural University, Taiyuan 030031, China; 20233788@stu.sxau.edu.cn (G.G.); 202430025@stu.sxau.edu.cn (M.S.); 202430029@stu.sxau.edu.cn (Y.X.)

**Keywords:** UAV, path planning, data collection, transformer, multi-agent deep reinforcement learning

## Abstract

Multi-agent path planning for Unmanned Aerial Vehicles (UAVs) in agricultural data collection tasks presents a significant challenge, requiring sophisticated coordination to ensure efficiency and avoid conflicts. Existing multi-agent reinforcement learning (MARL) algorithms often struggle with high-dimensional state spaces, continuous action domains, and complex inter-agent dependencies. To address these issues, we propose a novel algorithm, Multi-Agent Transformer-based Soft Actor–Critic (MATRS). Operating on the Centralized Training with Decentralized Execution (CTDE) paradigm, MATRS enables safe and efficient collaborative data collection and trajectory optimization. By integrating a Transformer encoder into its centralized critic network, our approach leverages the self-attention mechanism to explicitly model the intricate relationships between agents, thereby enabling a more accurate evaluation of the joint action–value function. Through comprehensive simulation experiments, we evaluated the performance of MATRS against established baseline algorithms (MADDPG, MATD3, and MASAC) in scenarios with varying data loads and problem scales. The results demonstrate that MATRS consistently achieves faster convergence and shorter task completion times. Furthermore, in scalability experiments, MATRS learned an efficient “task-space partitioning” strategy, where the UAV swarm autonomously divides the operational area for conflict-free coverage. These findings indicate that combining attention-based architectures with Soft Actor–Critic learning offers a potent and scalable solution for high-performance multi-UAV coordination in IoT data collection tasks.

## 1. Introduction

The widespread deployment of wireless sensor networks (WSNs) has become a cornerstone of the Internet of Things (IoT), enabling deep integration and application across various societal domains [1,2,3]. A particularly impactful application is in the domain of smart agriculture, where the deployment of diverse sensor nodes across farmlands allows for the real-time acquisition of fine-grained operational data [4,5,6]. This capability is crucial to enabling the dynamic monitoring of crop growth statuses and providing a scientific basis for precision management practices, such as targeted fertilization and irrigation.

However, efficiently collecting data from sensor nodes dispersed across vast agricultural fields remains a significant challenge, particularly in remote areas with limited network infrastructure [7,8]. To address this, employing mobile devices to assist in data collection has emerged as a viable strategy. Among the available mobile platforms, Unmanned Aerial Vehicles (UAVs) offer a uniquely flexible, efficient, and cost-effective solution for data gathering from WSNs compared with their ground-based counterparts [9,10].

The application of UAVs for data acquisition has given rise to a rich body of literature focused on optimization. Depending on mission objectives, research efforts have targeted distinct performance metrics, such as minimizing task completion time, system energy consumption, the Age of Information (AoI), and network coverage quality [11,12,13,14,15].

A primary branch of this research field focuses on optimizing the data collection trajectory for a single UAV. For instance, in agricultural scenarios, the authors in [16] employ a grid-based method to partition the area and select cluster heads for data aggregation, subsequently utilizing a Competitive Double Deep Q-Network (Competitive Double DQN) to optimize the UAV’s traversal path, thereby improving both collection efficiency and coverage quality. Addressing a similar agricultural setting, the study in [17] extends this by optimizing MAC layer mechanisms, introducing packet prioritization and timeslot allocation schemes to achieve more efficient resource utilization. In more complex urban environments, researchers in [18] investigate UAV data collection in a 3D space with signal jammers and obstacles, leveraging a Double Deep Q-Network (DDQN) to enhance the UAV’s task execution capabilities.

As IoT deployment scenarios expand, single-UAV systems face inherent limitations in efficiency and coverage, making cooperative multi-UAV path planning a prominent research hotspot. Exemplifying this trend, the authors of [19] proposed a multi-agent reinforcement learning (MARL) framework that integrates Double Deep Q-Network (DDQN), SARSA, and Q-learning algorithms. This hybrid approach enables effective cooperative path planning, leading to notable improvements in data collection efficiency and reductions in system energy consumption. Building upon this, system adaptability was enhanced in [20] by structuring the operational scene as a three-dimensional vector and introducing a convolutional layer into the DDQN architecture. Notably, these studies are predicated on discrete action spaces, which can constrain UAV maneuverability and limit optimal performance in practical scenarios.

To overcome these limitations, a significant line of research has shifted towards multi-agent optimization within continuous action spaces. For example, the work in [21] addresses the IoT data collection task by proposing the MCDDPG-EPM algorithm, based on the Multi-Critic Deep Deterministic Policy Gradient (MCDDPG) framework, to minimize path length under battery constraints. Similarly, the authors in [22] focus on optimizing the AoI by employing an improved Particle Swarm Optimization (PSO) algorithm to fine-tune an MATD3 neural network.

Furthermore, to transcend the limitations of conventional reinforcement learning, architectural innovations have been introduced. Hierarchical reinforcement learning (HRL), for instance, has tackled long-term path planning for UAVs requiring mid-mission charging by decoupling macro-level tasks from micro-level actions [23]. In parallel, attention mechanisms have been integrated into network architectures, empowering models to dynamically focus on salient information, proving effective for scheduling multi-UAV systems in dynamic scenarios [24]. Collectively, these advancements underscore that innovation within the algorithmic architecture itself is a key avenue for enhancing MARL systems’ capabilities.

Recent advances have also explored Transformer-enhanced MARL frameworks for UAV applications. For example, self-attention mechanisms have been employed to coordinate multi-UAV air transportation and navigation tasks by capturing long-range inter-agent dependencies, resulting in more globally coherent route planning [25]. Similarly, Transformer-based critics have been applied to UAV-enabled communication networks, where attention-driven policies improve channel allocation efficiency and training stability [26]. These developments highlight a growing trend toward leveraging attention architectures to strengthen the representational capacity of MARL models.

Despite these advances, many widely used CTDE-based algorithms—such as MADDPG, MATD3, and MASAC—still depend on centralized critics that encode the joint state–action space via simple feature concatenation. Such a representation is fundamentally limited in its ability to model complex inter-agent relationships, often leading to unstable training, slow convergence, and suboptimal performance as the number of UAVs or sensor nodes increases. Motivated by these limitations, this work introduces a Transformer-based centralized critic that uses multi-head self-attention to explicitly capture inter-agent interactions, thereby enabling more expressive Q-value estimation, faster convergence, and improved scalability.

Building on the foregoing discussion, this paper addresses the challenge of cooperative data collection from IoT sensor nodes using a multi-UAV system for agricultural applications. The primary contributions of this work are as follows:

1. This paper proposes a framework based on the MATRS algorithm designed for multi-UAV collaborative data collection. The multi-agent data collection problem is modeled as a decentralized Partially Observable Markov Decision Process (Dec-POMDP), and a solution based on MASAC (Multi-Agent Soft Actor–Critic) is presented. The framework adopts a Centralized Training with Decentralized Execution (CTDE) paradigm, which ensures autonomous decision-making capability for each UAV while simultaneously enabling the learning of efficient global collaborative strategies.
2. Integration of a Transformer architecture to enhance inter-agent collaboration. Diverging from conventional coordination mechanisms, this work incorporates a Transformer encoder within the critic network of the MASAC framework. By leveraging the self-attention mechanism, our approach enables the system to dynamically weight the importance of information from different agents, effectively capturing long-range, global spatiotemporal dependencies. This facilitates a more sophisticated and far-sighted form of cooperative decision making, particularly in scheduling data collection tasks among UAVs.
3. Comprehensive experimental validation and scalability analysis in a smart agriculture scenario. We conducted rigorous experiments in a simulated agricultural environment, benchmarking the proposed MATRS algorithm against several state-of-the-art baseline methods. The empirical results demonstrate that MATRS achieves superior performance, exhibiting a significantly higher data collection rate and a shorter mission completion time. Furthermore, by evaluating the algorithm across various problem scales, we confirm its robustness and excellent scalability.

The remainder of this paper is organized as follows: In Section 2, we outline the system model and provide the formal problem formulation. Section 3 details the design and implementation of the proposed MATRS algorithm. In Section 4, we present the simulation experiments conducted to evaluate the UAV data collection paths and discuss the corresponding results. Finally, Section 5 concludes the paper.

## 2. System Model

**Figure 1:**
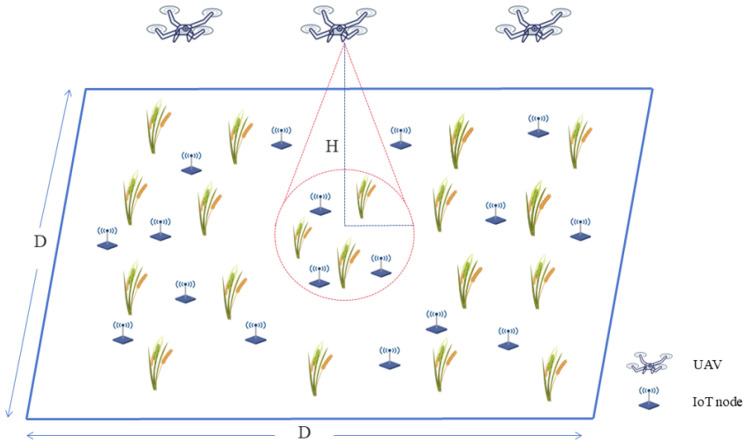
System model for multi-UAV data collection. The scenario involves *K* UAVs flying at a fixed altitude *H* over a D×D agricultural area containing *N* distributed sensor nodes. The cone represents the UAV’s communication footprint, within which data transmission is established.

We consider a multi-UAV cooperative data collection scenario, as illustrated in Figure 1. The operational environment is defined as a three-dimensional agricultural area of size D×D×H.

Within this area, a set $S=\{s_{1},s_{2},\ldots ,s_{N}\}$ of *N* static sensor nodes is deployed. The position of each sensor node $s_{n}\in S$ is fixed and known, with its coordinates given by $q_{n}=\left(x_{n},y_{n},0\right)$.

The data collection task is executed by a fleet of *K* homogeneous UAVs, denoted by the set $U=\{u_{1},u_{2},\ldots ,u_{K}\}$. To simplify the analysis and focus on horizontal trajectory optimization, all UAVs are assumed to maintain a constant flight altitude of *H*. This altitude is typically chosen to balance communication coverage area with signal quality and to ensure safe clearance above crops.

The total mission duration is denoted by *T* (in seconds). For the purpose of decision making, we discretize this period into a sequence of *L* time slots, where each slot has a uniform duration of $\delta_{t}$ (in seconds). The set of time slots is thus defined as 𝒯={0,1,…,L−1}, with $L=T/\delta_{t}$.

In this discrete-time model, the instantaneous position of UAV $u_{k}$ in any time slot t∈𝒯 is uniquely determined by its two-dimensional horizontal coordinates, $p_{k}\left(t\right)=\left(x_{k}\left(t\right),y_{k}\left(t\right)\right)$. Consequently, the individual trajectory of UAV $u_{k}$ throughout the entire mission, denoted by $P_{k}$, can be defined as the sequence of its positions in each time slot:

(1) $$\Gamma_{k}=\left\{p_{k}\left(0\right),p_{k}\left(1\right),\ldots ,p_{k}\left(L-1\right)\right\}$$

Correspondingly, the joint trajectory of the entire UAV fleet, 𝒫𝒫, is the collection of all individual trajectories:

(2) $$G=\{\Gamma_{1},\Gamma_{2},\ldots ,\Gamma_{K}\}$$

### 2.1. UAV Energy Consumption Model

The total energy consumption during the mission comprises two primary components: the propulsion energy required for flight and the communication energy for data transmission with the sensor nodes. In the data collection task we investigate, a communication link is established only when the received signal power between a UAV and a sensor node exceeds a predefined threshold. During these brief data exchange intervals, the energy consumed for communication is negligible compared with the energy required for propulsion.

Therefore, in modeling the mission’s energy consumption, we focus exclusively on the dominant component: the flight energy. Following the widely used rotary-wing UAV energy model proposed by Zeng et al. [27], the power consumption P(v) (in Watts) for a UAV flying at a speed *v* (in m/s) can be modeled as

(3) $$P\left(v\right)=P_{0}\left(1+\frac{3v^{2}}{U_{\mathrm{tip}}^{2}}\right)+\frac{1}{2}d\rho \delta Av^{3}+P_{i}{\left(\sqrt{1+\frac{v^{4}}{4v_{0}^{4}}}-\frac{v^{2}}{2v_{0}^{2}}\right)}^{\frac{1}{2}}$$

The parameters in Equation (3) are defined as follows: $P_{0}$ (Watts) and $P_{i}$ (Watts) are constants representing the blade profile power and induced power in hovering state, respectively; $U_{\mathrm{tip}}$ (m/s) is the tip speed of the rotor blade; $v_{0}$ (m/s) is the mean rotor-induced velocity in hover; *d* and δ denote the fuselage drag ratio and rotor solidity; and ρ (kg/m^3^) and *A* (m^2^) represent the air density and the rotor disc area, respectively.

Based on this power model, the total flight energy consumption $E_{k}$ (in kJ) of UAV $u_{k}$ over the entire mission can be calculated by summing its power consumption in each time slot:

(4) $$E_{k}=\sum_{t=0}^{L-1}P\left(v_{k}\left(t\right)\right)\cdot \delta_{t}$$

where $v_{k}\left(t\right)$ is the speed of UAV $u_{k}$ in time slot *t*. The total energy consumption of the entire UAV fleet, $E_{\mathrm{total}}$ (kJ), is the sum of the individual energy consumption of all UAVs:

(5) $$E_{\mathrm{total}}=\sum_{k=1}^{K}E_{k}$$

Furthermore, to ensure that all UAVs can successfully complete the mission, each UAV must adhere to its own energy constraint. Assuming that each UAV starts with an initial energy budget of $E_{\max}$ (in kJ), the cumulative energy consumed by any UAV $u_{k}$ must not exceed this budget at any point during the mission. This constraint can be formally expressed as follows: For any UAV *k* and any time slot $t^{\prime }$, the following inequality must hold:

(6) $$\sum_{t=0}^{t^{\prime }}P\left(v_{k}\left(t\right)\right)\cdot \delta_{t}\leq E_{\max},\;\;\;\;\forall k\in \left\{1,\;\ldots ,K\right\},\forall t^{\prime }\in \left\{0,\ldots ,L-1\right\}$$

### 2.2. Channel Model

In agricultural data collection scenarios, the wireless channel between a UAV and a sensor node is highly dependent on the type and height of the crops. For analytical tractability, we base our channel model on the Line-of-Sight (LoS) propagation assumption, which is generally valid for environments with low-stalk crops or where sensor modules are elevated above the vegetation canopy.

To better reflect the practical conditions of an agricultural field, we augment the standard free-space path loss model with an additional, constant attenuation factor, $L_{\mathrm{veg}}$, to account for the signal loss caused by vegetation. Consequently, the average path loss $PL_{k,n}$ between UAV *k* at position $p_{k}\left(t\right)$ and sensor node *n* is given by the modified Friis equation in decibels:

(7) $$PL_{k,n}\left(t\right)=20\log_{10}\left(d_{k,n}\left(t\right)\right)+20\log_{10}\left(f\right)+20\log_{10}\left(\frac{4\pi }{c}\right)+L_{\mathrm{veg}}$$

where $d_{k,n}\left(t\right)=∥p_{k}\left(t\right)-q_{n}∥$ (meters) is the Euclidean distance between the UAV and the sensor node in time slot *t*, *f* (Hz) is the carrier frequency, *c* (m/s) is the speed of light, and $L_{\mathrm{veg}}$ (dB) is the additional loss component due to vegetation.

It is important to acknowledge the limitations of this model. In environments with dense or tall crops (e.g., cornfields), signal propagation can be significantly affected by scattering, diffraction, and absorption, leading to complex Non-Line-of-Sight (NLoS) conditions. While our inclusion of $L_{\mathrm{veg}}$ provides a first-order approximation, a more sophisticated, probabilistic channel model might be required for such scenarios. However, for the scope of this work, the augmented LoS model provides a reasonable and computationally tractable basis for trajectory optimization, particularly in typical precision-agriculture deployment that involves low-stalk crops or utilizes elevated sensor modules to maintain approximate LoS connectivity.

Building upon the path loss model from Equation (7), the received signal power $P_{\mathrm{rx},k,n}\left(t\right)$ (in dBm) at the UAV *k* from sensor node *n* in time slot *t* can be expressed as

(8) $$P_{\mathrm{rx},k,n}\left(t\right)=P_{\mathrm{tx}}-PL_{k,n}\left(t\right)$$

where $P_{\mathrm{tx}}$ (in dBm) is the constant transmission power of the sensor nodes. We adopt a transmission power of $P_{\mathrm{tx}}=20$ dBm, as this choice is not only consistent with prior studies [21,27] but also represents a typical power level for nodes in modern wireless sensor networks.

In practice, a stable communication link can only be established if the received signal power is above the UAV’s minimum reception sensitivity. To model this requirement, we introduce a reception power threshold, $P_{\mathrm{th}}$ (in dBm). Therefore, for UAV *k* to effectively communicate with sensor node *n*, its received power must satisfy the following constraint:

(9) $$P_{\mathrm{rx},k,n}\left(t\right)\geq P_{\mathrm{th}}$$

This constraint implies that data collection is only feasible when a UAV is sufficiently close to a sensor node, such that the path loss is below a certain maximum. If this condition is not met, the channel between them is considered unavailable, and the data rate is effectively zero.

Assuming that the channel is subject to Additive White Gaussian Noise (AWGN) with constant power spectral density, resulting in a noise power of $\sigma^{2}$ (in Watts), the Signal-to-Noise Ratio (SNR) ${\mathrm{SNR}}_{k,n}\left(t\right)$ at UAV *k* from sensor node *n* can be expressed as

(10) $${\mathrm{SNR}}_{k,n}\left(t\right)=\frac{S_{k,n}\left(t\right)}{\sigma^{2}}=\frac{10^{\frac{P_{\mathrm{rx},k,n}\left(t\right)-30}{10}}}{\sigma^{2}}$$

where $S_{k,n}\left(t\right)$ is the received power in linear scale (Watts), converted from the logarithmic scale (dBm) representation $P_{\mathrm{rx},k,n}\left(t\right)$.

Finally, according to the Shannon–Hartley theorem, the maximum achievable data rate $R_{k,n}\left(t\right)$ (in bits per second) over a channel with bandwidth *B* (in Hz) is given by

(11) $$R_{k,n}\left(t\right)=B\log_{2}\left(1+{\mathrm{SNR}}_{k,n}\left(t\right)\right)$$

### 2.3. Problem Formulation

Based on the system model detailed above, the central task of this research study can be formulated as a multi-objective optimization problem (MOOP). The goal is to jointly optimize the trajectories of the UAV fleet, 𝒫, and the mission completion time, $T_{c}$, to simultaneously maximize the total data throughput while minimizing the completion time, all subject to the operational constraints previously defined.

The two conflicting objectives are formally stated as follows:

(Objective 1: Maximize Total Throughput)

(12) $$\max_{G,T_{c}}\;\;\;\;F_{1}=\sum_{t=0}^{\lfloor T_{c}/\delta_{t}\rfloor -1}\sum_{k=1}^{K}\sum_{n=1}^{N}R_{k,n}\left(t\right)\cdot \delta_{t}$$

(Objective 2: Minimize Completion Time)

(13) $$F_{2}=T_{c}\min_{G,T_{c}}\;\;\;\;F_{2}=T_{c}$$

## 3. Path Planning Based on Deep Reinforcement Learning

### 3.1. Multi-Agent POMDP Formulation

In the preceding section, we formulated the multi-UAV cooperative data collection mission as a complex optimization problem. This problem is characterized by the involvement of multiple autonomous agents operating within an environment defined by a high-dimensional state space, a continuous action space, and intricate inter-agent dependencies. These characteristics render traditional optimization algorithms computationally intractable or ineffective.

As noted in the UAV motion-planning literature [28], this work focuses specifically on path planning, that is, generating collision-free and task-oriented routes for each UAV on a discrete time horizon. The resulting paths are required to satisfy only a set of basic dynamic-feasibility constraints, such as bounded velocity and limits on heading-angle changes, rather than a full time-continuous trajectory planning formulation involving detailed kinematic or dynamic parameterization. Framed as a sequential decision-making problem under these conditions, the task is exceptionally well-suited for a multi-agent reinforcement learning approach.

Reinforcement learning (RL) presents a powerful paradigm for solving such complex sequential decision-making problems [29]. The core principle of RL is that agents learn an optimal policy not from a pre-specified model of the environment’s dynamics but through a process of trial-and-error interaction. By continuously exploring the environment and receiving feedback in the form of rewards, agents gradually refine their strategies to maximize a long-term cumulative reward. Furthermore, for sequential decision problems where agents have access to only a fraction of the full environmental state, the Partially Observable Markov Decision Process (POMDP) framework offers an ideal modeling tool [30,31,32].

Therefore, to address our optimization problem, this paper proposes the Multi-Agent Transformer-based Soft Actor–Critic (MATRS) algorithm, an advancement upon the foundational MASAC algorithm. Specifically, we model the multi-UAV data collection task as a POMDP, which is formally defined by the tuple 〈𝒮,𝒪,𝒜,𝒫,ℛ,γ〉. In this tuple, 𝒮 represents the global state space of the environment; 𝒪 is the joint observation space for all UAV agents; 𝒜 is the joint action space; 𝒫 denotes the state transition function, which defines the probability of transitioning from state *s* to $s^{\prime }$ after taking joint action *a*; ℛ is the reward function that provides a scalar feedback signal; and γ∈[0,1) is the discount factor, which balances the trade-off between immediate and future rewards.

### 3.2. Observation and Action Spaces

#### 3.2.1. Observation Space

In any given time slot *t*, the local observation of each UAV agent $u_{k}$ is a vector $o_{k}^{t}$ that encapsulates its own state and its perception of the environment. The components of this observation vector are detailed as

(14) $$o_{k}^{t}=\left[\underset{\mathrm{Proprioceptive}\;\mathrm{State}}{\underset{︸}{p_{k}\left(t\right),v_{k}\left(t\right),\theta_{k}\left(t\right),C_{k}\left(t\right)}},\underset{\mathrm{Exteroceptive}\;\mathrm{Perception}}{\underset{︸}{q_{1},D_{k,1}\left(t\right),\ldots ,q_{N},D_{k,N}\left(t\right)}}\right]$$

where the elements are defined as follows:

- $p_{k}\left(t\right)$: The 2D Cartesian coordinates $\left(x_{k}\left(t\right),y_{k}\left(t\right)\right)$ of UAV *k*, representing its current position.
- $v_{k}\left(t\right)$: The current speed of UAV *k*.
- $\theta_{k}\left(t\right)$: The current heading angle of UAV *k*.
- $C_{k}\left(t\right)$: A boolean flag indicating if the safety controller was triggered for UAV *k* in the previous time step (e.g., due to a potential collision or boundary violation). A value of 1 signifies activation.
- $q_{n}$: The fixed 2D coordinates of sensor node $s_{n}$. This is repeated for all *N* sensors.
- $D_{k,n}\left(t\right)$: The cumulative quantity of data (in bits) that UAV *k* has collected from sensor node $s_{n}$ up to the beginning of time slot *t*.

The dimension of the local observation vector is, therefore, $o_{\mathrm{local}}=5+3N$, where *N* is the number of sensor nodes. The joint observation space for all agents in time slot *t*, denoted by ${𝒪}^{t}$, is the set of all individual observations: ${𝒪}^{t}=\left\{o_{1}^{t},o_{2}^{t},\ldots ,o_{K}^{t}\right\}$.

#### 3.2.2. Action Space

To enable flexible and fine-grained control within the complex operational environment, we define a continuous action space for each UAV agent. In each time slot *t*, the action $a_{k}\left(t\right)$ for UAV *k* is a two-dimensional vector:

(15) $$a_{k}\left(t\right)=\left[a_{v},a_{\theta }\right]$$

The components of this action vector are defined as follows:

- $a_{v}\in \left[-1,1\right]$ is the normalized acceleration command. This value is scaled to a physical acceleration value to adjust the UAV’s flight speed for the next time step.
- $a_{\theta }$ is the commanded change in the UAV’s heading angle. To respect the physical maneuverability limits of the UAV, this change is constrained. Specifically, the change in heading angle between two consecutive time steps cannot exceed 1.2 radians, meaning that $a_{\theta }\in \left[-1.2,1.2\right]$ rad.

Thus, each agent has a local action space of dimension $a_{\mathrm{local}}=2$. The joint action space ${𝒜}^{t}$ in time slot *t* is the set of all individual actions: ${𝒜}^{t}=\left\{a_{1}\left(t\right),a_{2}\left(t\right),\ldots ,a_{K}\left(t\right)\right\}$.

### 3.3. Reward Function Design

A central challenge in applying multi-agent reinforcement learning to the cooperative data collection task is the problem of sparse rewards. If a reward is provided only upon final mission completion, agents face a severe credit assignment problem: they struggle to attribute the final outcome to specific actions taken over a long sequence of steps. This lack of immediate, informative feedback drastically hinders the learning process, often causing the agents to converge to suboptimal policies that fail to complete the full data collection task, resulting in poor convergence and incomplete outcomes.

To overcome this challenge and to effectively guide the agents towards a globally optimal cooperative strategy, we have designed a comprehensive, multi-component reward function. This function decomposes the high-level mission objective into a set of immediate, dense reward and penalty signals. The total reward for each agent *k* at time step *t*, denoted by $R_{k}\left(t\right)$, is a weighted sum of five distinct components:

(16) $${ℛ}_{k}\left(t\right)=r_{\mathrm{safety}}\left(t\right)+r_{\mathrm{guide}}\left(t\right)+r_{\mathrm{collect}}\left(t\right)+r_{\mathrm{comp}}\left(t\right)+r_{\mathrm{step}}\left(t\right)$$

Each component is detailed below.

(a) Safety Penalty ($r_{\mathrm{safety}}$)

To ensure safe operation, a significant penalty is imposed for any violation of operational boundaries or inter-agent safety distances. The safety controller flag $C_{k}\left(t\right)$ is set to 1 if the distance between UAV *k* and any other UAV falls below a safety threshold $d_{\mathrm{safe}}$ or if UAV *k* moves outside the predefined operational area. Otherwise, $C_{k}\left(t\right)$ is 0.

(17) $$r_{\mathrm{safety}}\left(t\right)=\left\{\begin{array}{cc} -10 & \mathrm{if}C_{k}\left(t\right)=1\\ 0 & \mathrm{if}C_{k}\left(t\right)=0 \end{array}\right.$$

(b) Guidance Reward ($r_{\mathrm{guide}}$)

To improve exploration efficiency, a dense guidance reward steers the UAVs purposefully. A small positive reward is given for approaching an un-serviced node, while a penalty is applied for deviating from the task area.

(18) $$r_{\mathrm{guide}}\left(t\right)=\left\{\begin{array}{cc} +0.5 & \mathrm{if}\;\mathrm{approaching}\;\mathrm{an}\;\text{un-serviced}\;\mathrm{node}\\ -5.0 & \mathrm{if}\;\mathrm{deviating}\;\mathrm{from}\;\mathrm{the}\;\mathrm{task}\;\mathrm{area}\\ 0 & \mathrm{otherwise} \end{array}\right.$$

(c) Data Collection Reward ($r_{\mathrm{collect}}$)

To directly incentivize task progress, a reward is granted proportional to the amount of data collected in the current time step.

(19) $$r_{\mathrm{collect}}\left(t\right)=50\cdot \frac{\Delta D_{k}\left(t\right)}{D_{\mathrm{unit}}}$$

where $\Delta D_{k}\left(t\right)$ denotes the data collected by UAV *k* in time slot *t* and $D_{\mathrm{unit}}$ is a normalization constant. In this work, $D_{\mathrm{unit}}$ is defined as one-fifth of the total data capacity of a single sensor node, serving as the normalization unit for data collection rewards.

(d) Task Completion Reward ($r_{\mathrm{comp}}$)

A large, one-time terminal reward is granted to all agents upon successful completion of the entire mission. This occurs in the exact time step in which the total required data are collected.

(20) $$r_{\mathrm{comp}}\left(t\right)=\left\{\begin{array}{cc} +200, & \mathrm{if}D_{\mathrm{collected}}\left(t\right)=D_{\mathrm{total}}\;\mathrm{and}\;D_{\mathrm{collected}}\left(t-1\right)<D_{\mathrm{total}}\\ 0, & \mathrm{otherwise} \end{array}\right.$$

where $D_{\mathrm{collected}}\left(t\right)$ denotes the total system-wide data collected up to time *t* and $D_{\mathrm{total}}$ is the overall quantity of data required to be collected.

(e) Step Penalty ($r_{\mathrm{step}}$)

Finally, to encourage time efficiency, a small, constant penalty is applied in every time step, implicitly motivating agents to complete the mission as quickly as possible.

(21) $$r_{\mathrm{step}}\left(t\right)=-0.1$$

### 3.4. MATRS Algorithm Architecture

#### 3.4.1. Centralized Training with Decentralized Execution (CTDE)

**Figure 2:**
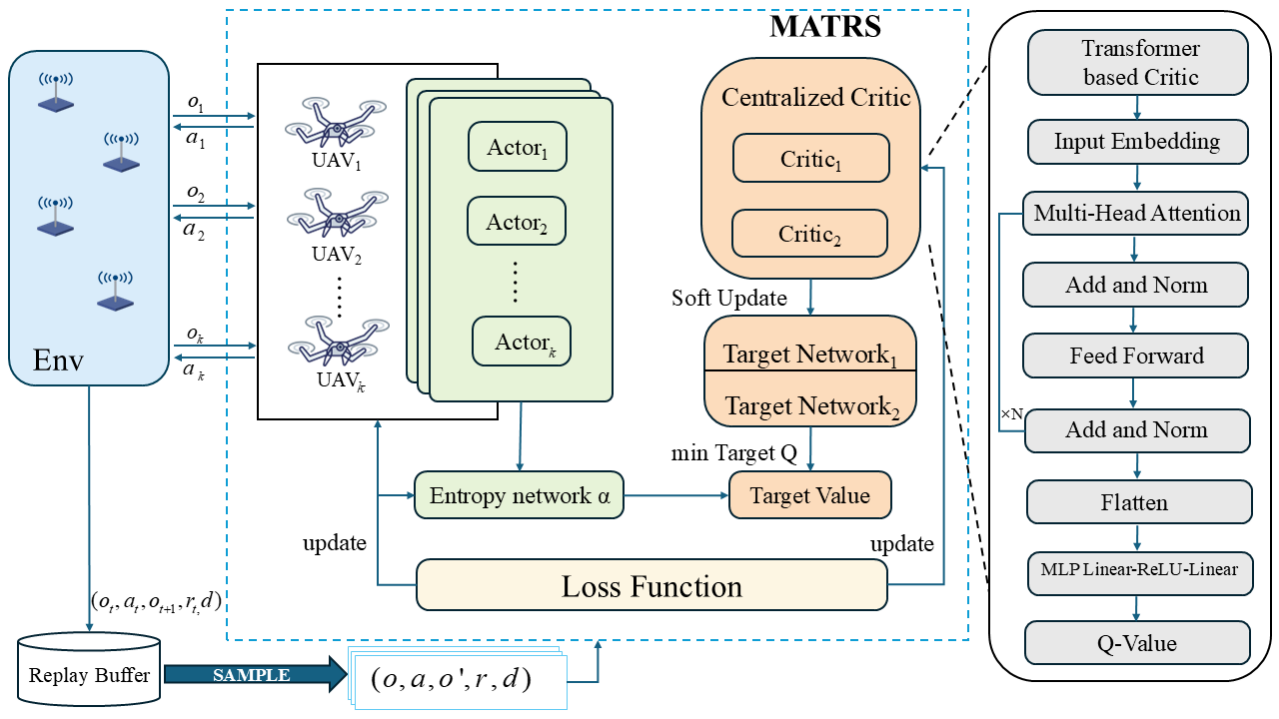
The architecture of the MATRS algorithm.

As illustrated in Figure 2, the MATRS algorithm adopts the widely used Centralized Training with Decentralized Execution (CTDE) framework. During the execution phase, each UAV agent *k* operates using an individual actor (policy) network, denoted by $\pi_{\phi_{k}}$.

In a significant departure from the approach in [33], which feeds global state information into the actor networks of a MASAC algorithm, the proposed MATRS algorithm adheres strictly to the principle of decentralized execution. Specifically, in each time slot *t*, agent *k* uses only its local observation $o_{k}\left(t\right)$ as the input to its actor network. The network’s output is the action $a_{k}\left(t\right)$ that the agent will execute in that time slot.

After all agents have executed their joint action $a\left(t\right)=\{a_{1}\left(t\right),\ldots ,a_{K}\left(t\right)\}$, the environment transitions and returns a set of feedback for each agent: its individual reward $r_{k}\left(t\right)$, its local observation for the next time slot $o_{k}\left(t+1\right)$, and a boolean flag *d* indicating mission termination. This complete interaction experience, structured as a transition tuple (o(t),a(t),r(t),o(t+1),f), is then stored in a shared experience replay buffer, 𝒟𝒟, for subsequent centralized training.

#### 3.4.2. Transformer-Based Centralized Critic Network

The core innovation of the MATRS algorithm lies in its centralized critic architecture, $Q_{\omega }\left(o,a\right)$. While traditional MASAC algorithms typically process agent information by simply concatenating all individual observations and actions into a single “flattened” vector, this approach discards the inherent structure of the multi-agent system and struggles to capture complex inter-agent dependencies.

To overcome this limitation, the MATRS critic integrates a Transformer Encoder to explicitly model these relationships. During the forward pass, each agent’s local observation $o_{k}$ and corresponding action $a_{k}$ are first concatenated to form an individual state–action feature vector. This vector is then passed through a linear projection layer to create an initial high-dimensional embedding, $e_{k}$, which serves as the token representation for agent *k*. Next, the embeddings of all agents, $\left\{e_{1},e_{2},\ldots ,e_{K}\right\}$, are assembled into a sequence and fed into a multi-layer Transformer Encoder. Within each layer, the multi-head self-attention mechanism dynamically computes the dependency weights among all agents, allowing the network to effectively capture complex interaction patterns and the global context of the joint state–action. Finally, the output embeddings from the Transformer are flattened and passed through a multi-layer perceptron (MLP) head, which maps these high-level features to a single scalar Q-value for robust value estimation. By leveraging self-attention, the MATRS critic learns a rich, structured representation of agent interactions, leading to more accurate value estimation than simple concatenation.

### 3.5. Complexity Analysis and Comparison

The primary architectural distinction between MATRS and MASAC lies in the adoption of a Transformer Encoder within the centralized critic. This structure introduces additional computational overhead compared with the MLP-based critic used in MASAC. The complexity of the Transformer Encoder arises from two components: the multi-head self-attention mechanism, whose computational cost scales quadratically with the number of agents *K*, and the position-wise feed-forward networks, whose cost scales linearly with *K* [34]. Consequently, the computational complexity of the MATRS critic is

(22) $$O(L_{\mathrm{tf}}\cdot \left(K^{2}d_{\mathrm{model}}+Kd_{\mathrm{model}}^{2}\right))$$

where $L_{\mathrm{tf}}$ denotes the number of Transformer layers and $d_{\mathrm{model}}$ is the internal embedding dimension.

In contrast, the MLP-based critic in MASAC has a substantially lower computational burden, characterized by

(23) $$O(K\left(o_{\mathrm{local}}+a_{\mathrm{local}}\right)d_{\mathrm{mlp}}+\left(L_{\mathrm{mlp}}-1\right)d_{\mathrm{mlp}}^{2})$$

where $L_{\mathrm{mlp}}$ is the number of network layers and $d_{\mathrm{mlp}}$ is the hidden dimension. The first term grows linearly with the number of agents *K*, while the second term remains independent of *K*, being determined solely by the depth and width of the network.

Overall, MATRS exhibits a clear quadratic complexity in the number of agents, whereas MASAC scales only linearly. This difference translates into a higher computational cost per training episode for MATRS. Table 1 summarizes the architectural differences and parameter sizes for both approaches, with all values computed for a scenario involving K=3 agents and N=15 sensor nodes.

### 3.6. Optimization and Training Updates

#### 3.6.1. Critic Network Update

The centralized critic networks in the MATRS algorithm are trained to learn an accurate joint action–value function, $Q_{\omega }\left(o,a\right)$, by minimizing the mean squared Bellman error (MSBE). The update process is rooted in the principles of Soft Actor–Critic (SAC) and incorporates the clipped double Q-learning technique to mitigate Q-value overestimation.

First, for a given transition $\left(o,a,r,o^{\prime },f\right)$ sampled from the replay buffer 𝒟, a target value *y* is computed. This involves maintaining two separate critic networks, $Q_{\omega_{1}}$ and $Q_{\omega_{2}}$, along with their corresponding slow-moving target networks, $Q_{\omega_{1}^{\prime }}$ and $Q_{\omega_{2}^{\prime }}$. The target value is defined as

(24) $$y=r+\gamma \left(1-f\right)\left(\min_{j=1,2}Q_{\omega_{j}^{\prime }}\left(o^{\prime },a^{\prime }\right)-\alpha \log\pi_{\phi }\left(a^{\prime }|o^{\prime }\right)\right)$$

The components of this target value are as follows:

- $r=\sum_{k=1}^{K}r_{k}$ is the sum of rewards for all agents.
- γ is the discount factor.
- *f* is the task completion flag.
- $a^{\prime }∼\pi_{\phi }\left(\cdot |o^{\prime }\right)$ is the joint action for the next observation $o^{\prime }$, sampled from the current policy.
- The entropy regularization term, weighted by the temperature α, encourages exploration.

Subsequently, each critic network’s parameters are updated independently by minimizing their respective mean squared error loss with respect to this common target *y*. The individual loss functions for the two critic networks, $Q_{\omega_{1}}$ and $Q_{\omega_{2}}$, are

(25) $$\begin{array}{cc} 𝓛(\omega_{1}) & =E_{(o,a,r,o^{\prime },f)∼𝒟}\left[{\left(Q_{\omega_{1}}\left(o,a\right)-y\right)}^{2}\right] \end{array}$$

(26) $$\begin{array}{cc} 𝓛(\omega_{2}) & =E_{(o,a,r,o^{\prime },f)∼𝒟}\left[{\left(Q_{\omega_{2}}\left(o,a\right)-y\right)}^{2}\right] \end{array}$$

Both sets of parameters, $\omega_{1}$ and $\omega_{2}$, are then updated by performing separate gradient descent steps on their respective losses, typically using an optimizer such as Adam.

#### 3.6.2. Target Network Update

To ensure the stability of the learning process, the parameters of the target critic networks, $\omega_{1}^{\prime }$ and $\omega_{2}^{\prime }$, are not updated via gradient descent. Instead, they are updated to slowly track the parameters of the main critic networks using Polyak averaging. After each training iteration for the main critics, the target network parameters are updated according to the following soft update rule:

(27) $$\omega_{j}^{\prime }\leftarrow \tau \omega_{j}+\left(1-\tau \right)\omega_{j}^{\prime }\;\;\;\mathrm{for}\;j=1,2$$

where τ∈[0,1] is the soft update rate (e.g., τ=0.005), a small hyperparameter that controls how quickly the target networks change. This process of using a slow-moving target network is critical to providing a stable target *y* during the calculation of the critic loss, thereby preventing divergent or oscillating training behavior.

#### 3.6.3. Actor and Temperature Update

The actor network, $\pi_{\phi }$, is updated with the objective of learning a policy that maximizes the weighted sum of the expected return and the policy entropy. The loss function for the actor is, therefore, designed to simultaneously encourage actions that lead to higher Q-values (as estimated by the critic) and maintain a high degree of exploration.

To achieve this, the actor parameters ϕ are optimized by minimizing the following loss function:

(28) $$𝓛\left(\phi \right)=E_{o∼𝒟,a∼\pi_{\phi }}\left[\alpha \log\pi_{\phi }\left(a|o\right)-\min_{j=1,2}Q_{\omega_{j}}\left(o,a\right)\right]$$

Minimizing this loss drives the policy $\pi_{\phi }$ to select actions that have higher estimated Q-values, while the entropy term $\alpha \log\pi_{\phi }\left(a|o\right)$ prevents premature convergence by encouraging exploration.

To balance exploration and exploitation automatically, the temperature parameter α is also learned by minimizing its own loss function. The goal is to adjust α such that the policy’s entropy remains close to a predefined target entropy, ℋ. The loss function for α is

(29) $$𝓛\left(\alpha \right)=E_{o∼𝒟,a∼\pi_{\phi }}\left[-\alpha \left(\log\pi_{\phi }\left(a|o\right)+ℋ\right)\right]$$

where ℋ is the target entropy, typically set to the negative of the action space dimension (i.e., ℋ=−dim(𝒜)), as recommended in the original SAC paper [35]. By optimizing this function, the algorithm automatically tunes α to maintain the desired level of policy stochasticity. The key steps of the MATRS training process are summarized in Algorithm 1.
**Algorithm 1** The MATRS training algorithm  1:**Initialize:**  2:For each agent k∈{1,…,K}, initialize actor network $\pi_{\phi_{k}}$ with random parameter $\phi_{k}$.  3:Initialize two critic networks $Q_{\omega_{1}},Q_{\omega_{2}}$ with random parameters $\omega_{1},\omega_{2}$.  4:Initialize target critic networks with $\omega_{1}^{\prime }\leftarrow \omega_{1}$, $\omega_{2}^{\prime }\leftarrow \omega_{2}$.  5:Initialize the temperature parameter α and the replay buffer 𝒟.  6:**for** episode $=1,\ldots ,M_{\mathrm{episodes}}$ **do**  7:   Reset environment and receive initial joint observation $o=\{o_{1},\ldots ,o_{K}\}$.  8:   **for** $t=1,\ldots ,T_{\max}$ **do**  9:       For each agent *k*, select action $a_{k}∼\pi_{\phi_{k}}\left(\cdot |o_{k}\right)$. {Decentralized execution}10:       Execute joint action $a=\{a_{1},\ldots ,a_{K}\}$ and observe rewards $r=\{r_{1},\ldots ,r_{K}\}$, next joint observation $o^{\prime }$, and done flag *d*.11:       Store the transition tuple $\left(o,a,r,o^{\prime },f\right)$ in the replay buffer 𝒟.12:       $o\leftarrow o^{\prime }$.13:       **if** |𝒟|>batch_size **then**14:           Sample a mini-batch of transitions ${\left\{\left(o_{i},a_{i},r_{i},o_{i}^{\prime },d_{i}\right)\right\}}_{i=1}^{N}$ from 𝒟.15:           {**— Critic Update —**}16:           Compute the target value $y_{i}$ for each transition using Equation (24).17:           Update critic parameters $\omega_{1},\omega_{2}$ by minimizing the losses $𝓛(\omega_{1})$ and $𝓛(\omega_{2})$ from Equations (25) and (26).18:           **if** t(modpolicy_delay)=0 **then**19:              {**— Actor and Temperature Update —**}20:              Update actor parameters $\phi_{k}$ for all agents by minimizing 𝓛(ϕ) from Equation (28).21:              Update temperature α by minimizing 𝓛(α) from Equation (29).22:              {**— Target Network Soft Update —**}23:              Update target critic networks for j=1,2:24:              $\omega_{j}^{\prime }\leftarrow \tau \omega_{j}+\left(1-\tau \right)\omega_{j}^{\prime }$ using Equation (27).25:           **end if**26:       **end if**27:       **if** *f* is true **then**28:           **break**29:       **end if**30:       **if** any UAV runs out of battery **then**31:           **break**32:       **end if**33:   **end for**34:**end for**

## 4. Simulation Setup and Results Analysis

### 4.1. Simulation Setup

**Figure 3:**
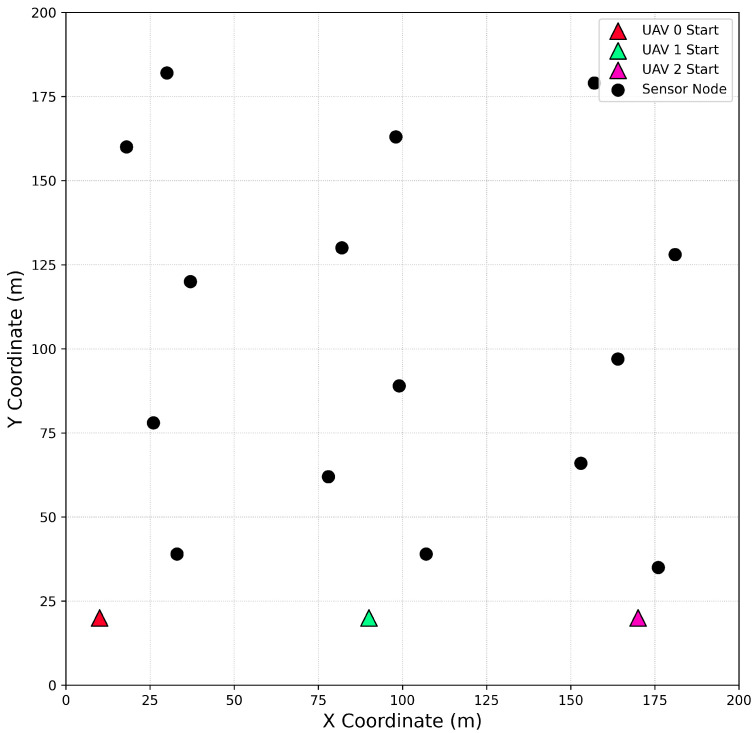
The benchmark simulation scenario, illustrating the initial positions of the 3 UAVs (triangles) and the 15 sensor nodes (circles) within the 200 m × 200 m operational area.

In this section, we conduct a series of simulation experiments to systematically evaluate the performance of the proposed MATRS algorithm under various scenarios. First, we construct a benchmark environment consisting of a 200 m × 200 m two-dimensional plane. Within this area, we deploy 15 sensor nodes and 3 UAVs. Each sensor node is initialized with 10 MB of data that needs to be collected. To ensure the reproducibility of our results, all UAVs start from fixed, predefined initial positions. The specific layout of this benchmark scenario is illustrated in Figure 3, where circular markers denote the locations of the sensor nodes and triangular markers indicate the starting positions of the UAVs.

To comprehensively evaluate the performance of the MATRS algorithm, we designed a set of comparative experiments along two key dimensions: scalability with respect to data load and scalability with respect to problem size.

First, to assess data load scalability, MATRS is benchmarked against three baseline algorithms: MADDPG, MATD3, and MASAC. The core objective of this comparison is to investigate how the performance of each algorithm evolves as the magnitude of the data collection task increases. To this end, we configured five experimental groups, setting the data volume to be collected from each sensor node to 10 MB, 20 MB, 30 MB, 40 MB, and 50 MB, respectively.

Second, to validate the algorithm’s feasibility and generalization capabilities in more complex environments, we evaluate its problem size scalability. We designed two additional scenarios: a medium-scale environment of 300 m × 300 m and a large-scale environment of 400 m × 400 m.

All key hyperparameters and environmental parameters used across these scenarios, such as those related to UAV–sensor communication, are detailed in Table 2.

To ensure the reliability and reproducibility of our findings, all simulation experiments were conducted on a unified high-performance computing platform. The platform was equipped with an Intel Core i7-12700K CPU (clocked at 5.0 GHz) and an NVIDIA GeForce RTX 4090 GPU, running on the Ubuntu 20.04 operating system. The simulation environment was built in Python (version 3.9.23), with the deep reinforcement learning models implemented using the PyTorch (version 2.7.1) framework.

The key hyperparameters used for training the MATRS algorithm and the baselines are detailed in Table 3. These parameters were kept consistent across all algorithms to ensure a fair comparison.

### 4.2. Results and Analysis

#### 4.2.1. Comparative Performance Analysis Under Varying Data Loads

**Figure 4:**
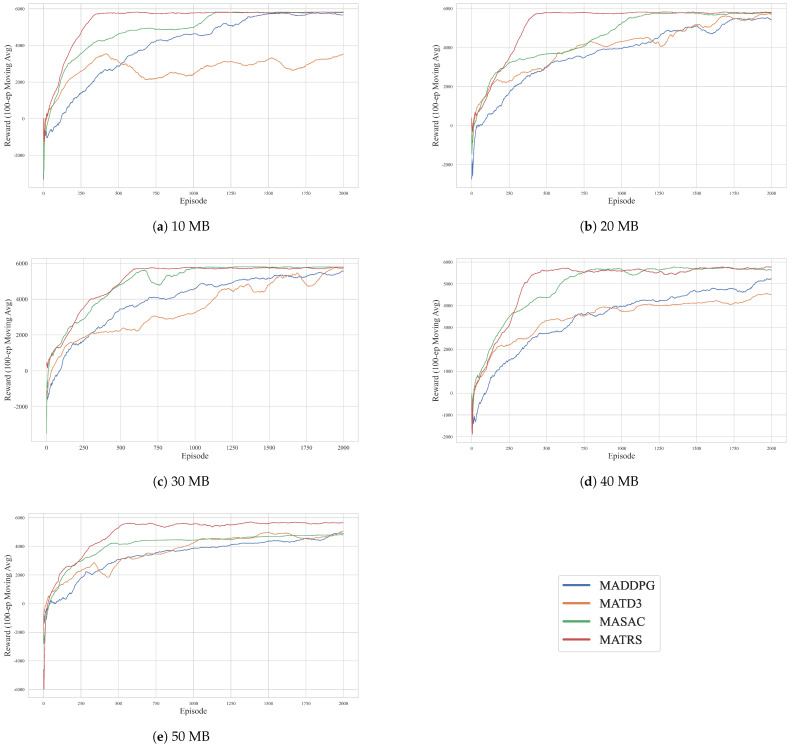
Learning curves of MATRS and baseline algorithms under varying data loads. The plots show the 100-episode moving average reward for scenarios with (**a**) 10 MB, (**b**) 20 MB, (**c**) 30 MB, (**d**) 40 MB, and (**e**) 50 MB of data per sensor node. MATRS (red) consistently demonstrates the fastest convergence and highest final reward across all scenarios.

**Figure A1:**
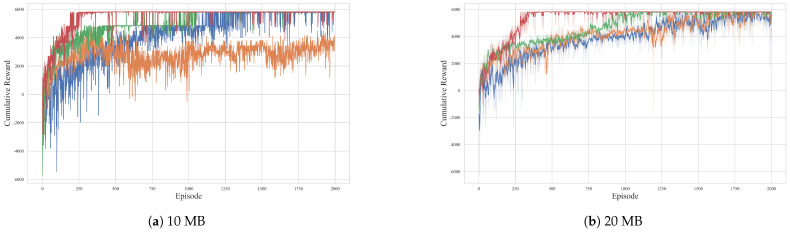

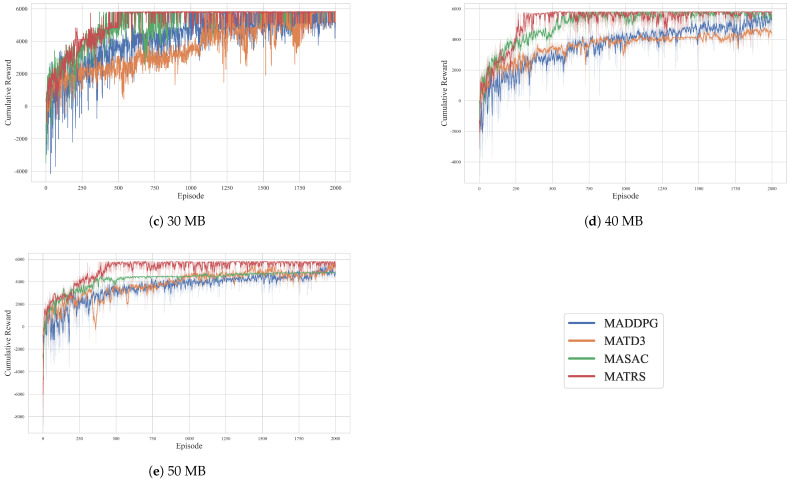
Cumulative reward per episode for MATRS and baseline algorithms under varying data loads.

To clearly evaluate the convergence trends and final performance stability of the compared algorithms, we adopt the 100-episode moving average reward as the primary evaluation metric. This metric smooths out short-term fluctuations and provides an intuitive representation of each algorithm’s learning efficiency. The results for the five different data load scenarios are presented in Figure 4. Appendix A Figure A1 presents the cumulative reward curves for all algorithms across five data load scenarios, offering additional insights into their performance.

As illustrated in Figure 4, the MATRS algorithm exhibits highly competitive convergence properties in all tested scenarios. Its learning curve is consistently among the steepest, enabling it to converge to the highest reward plateau with the greatest speed and stability. In the low-load 10 MB scenario, MATRS reaches its optimal policy in just around 250 episodes. While the number of episodes required for convergence increases slightly with the data load, MATRS remains highly efficient, stabilizing at the maximum reward within 500 episodes even in the high-load 50 MB scenario.

In contrast, the next-best performing algorithm, MASAC, demonstrates significantly slower convergence. Although it eventually reaches a high reward value, it typically requires 500 to 1000 episodes to approach a stable policy. Notably, the performance gap between MASAC and MATRS widens in the 50 MB scenario, further underscoring the advantages of the proposed method in high-demand situations.

The traditional DDPG-based algorithms, MADDPG and MATD3, perform poorly on this task. Their learning curves are characterized by severe oscillations and slow convergence throughout the training process. Furthermore, their final converged reward values are substantially lower than that of MATRS, especially under high data loads. This suggests that these baseline algorithms suffer from inefficient exploration, policy instability, and a tendency to become trapped in local optima when faced with multi-agent decision-making tasks involving complex operational constraints.

While the cumulative reward reflects overall learning performance, a more direct measure of an algorithm’s practical effectiveness is its task completion efficiency. We evaluate this using two key metrics: the data collection rate over time and the minimum steps required to complete the mission.

**Figure 5:**
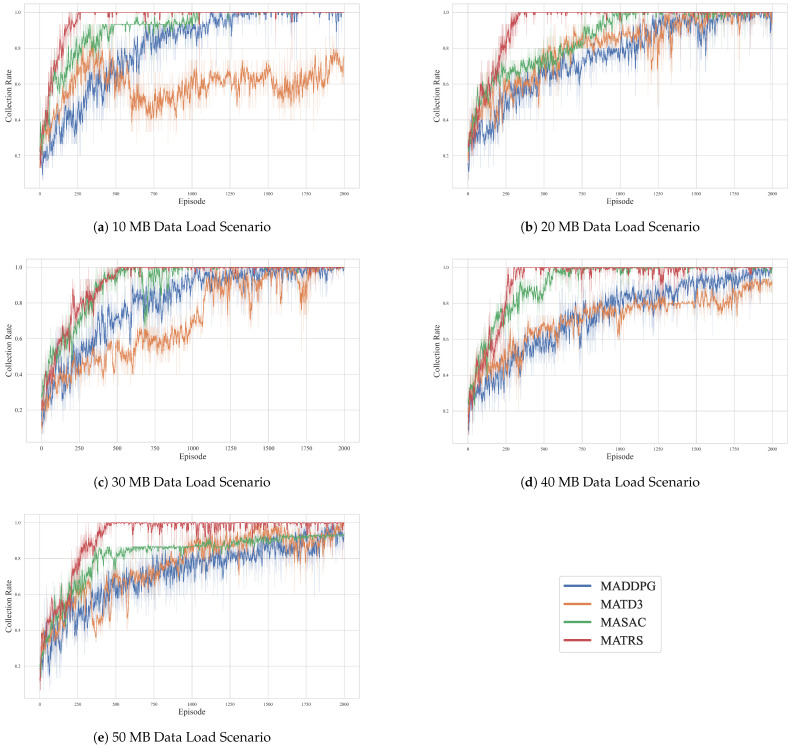
Data collection rate of MATRS and baseline algorithms under varying data loads. The plots compare the performance for five scenarios: (**a**) 10 MB, (**b**) 20 MB, (**c**) 30 MB, (**d**) 40 MB, and (**e**) 50 MB. The collection rate is a unitless ratio from 0 to 1. MATRS rapidly achieves and sustains a 100% collection rate across all scenarios.

The data collection rate, shown in Figure 5, highlights the overwhelming advantage of MATRS. It not only discovers a policy that achieves a 100% collection rate faster than all baselines but also maintains this optimal performance with minimal variance, demonstrating the stability of the learned policy.

To further quantify the efficiency of the final learned policies, we recorded the minimum number of steps each algorithm required to achieve 100% data collection across all data load scenarios. The results are summarized in Table 4. The data clearly indicate that MATRS is superior in both efficiency and stability. It consistently requires the fewest steps to complete the mission in all five scenarios. Furthermore, as the data load increases from 10 MB to 50 MB, the required steps for MATRS increase smoothly and predictably from 309 to 343. For instance, in the demanding 50 MB scenario, MATRS completes the task 147 steps faster than MASAC, which translates to a significant time saving of 14.7 s (at 0.1 s per step). This consistent efficiency and scalable performance demonstrate that the MATRS policy is exceptionally robust, adapting efficiently to increasing task difficulty.

In contrast, the baseline algorithms exhibit significant limitations. MATD3’s failure to complete the task in the 10 MB and 40 MB scenarios confirms its insufficient learning capability. While MADDPG and MASAC complete the task in most cases, the number of steps they require fluctuates erratically with increasing data load, indicating that their learned policies lack consistency and optimality.

Most revealing is the performance of MASAC. Despite its strong performance on the cumulative reward metric, it consistently requires the most steps among all successful algorithms. This exposes a critical flaw in the policy learned by MASAC: it converges to a high-reward yet time-inefficient local optimum. The policy learns to trade time for higher scores, likely by taking longer, risk-averse paths. This behavior is fundamentally at odds with the core mission objective of completing data collection as quickly as possible.

#### 4.2.2. Ablation Study on Critic Architecture

**Figure 6:**
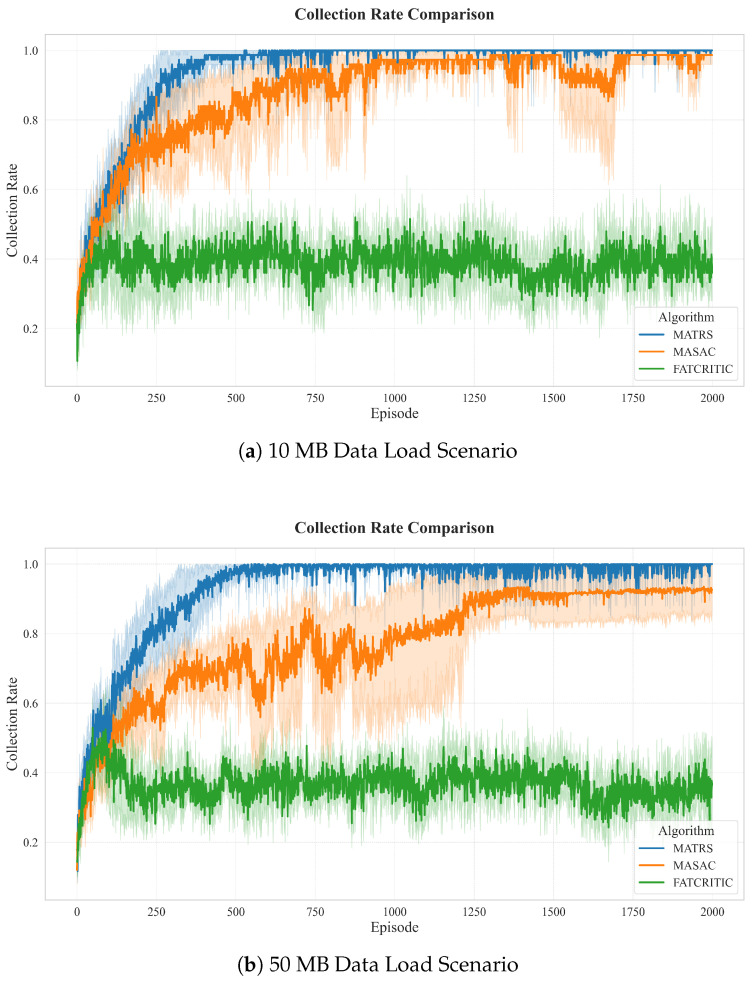
Ablation study results comparing MATRS, MASAC, and FATCRITIC. The plots show the data collection rate for the (**a**) 10 MB and (**b**) 50 MB scenarios. Solid lines represent the mean performance over 5 independent runs, while the shaded areas indicate the 95% confidence interval. FATCRITIC has a critic with a parameter count comparable to MATRS.

In this section, we introduce a new baseline algorithm, FATCRITIC, for comparative analysis. This method maintains an identical overall structure to MASAC, with the sole modification being the adoption of a wider and deeper MLP within its critic network. This is performed to scale its total parameter count to the same order of magnitude as the Transformer-based critic in MATRS. The purpose of this design is to eliminate the confounding factor of “disparity in model capacity”, thereby enabling a clearer examination of the contribution of the Transformer module itself. We conducted comparative training over five random seeds in two representative scenarios (10 MB and 50 MB data loads). The results are presented in Figure 6.

Across both scenarios, MATRS consistently achieves the best performance. Its learning curve is steeper, its convergence speed is significantly faster, and its final performance is the highest. Furthermore, its narrow confidence interval indicates strong robustness and stability across different random seeds. In comparison, MASAC is clearly inferior to MATRS in both learning speed and stability, which aligns with the findings from our previous comparative experiments.

Most revealingly, FATCRITIC fails to exhibit any effective learning trend in either scenario. Its collection rate stagnates around the 0.4 mark throughout the training process, with no discernible improvement, indicating a failure to learn a coordinated multi-UAV collection strategy. Despite having a parameter count comparable to MATRS, it lacks the expressive power to model inter-agent relationships.

This result strongly suggests that in multi-agent reinforcement learning, simply increasing the number of network parameters does not guarantee a performance improvement; the critical factor is the architectural design itself. The advantage of MATRS appears to originate not from being a “larger model” but from the Transformer Encoder’s intrinsic ability to effectively model the dynamic relationships between agents, making it significantly more effective than traditional MLP-based critics in this context.

#### 4.2.3. Scalability Analysis Across Different Problem Sizes

To evaluate the scalability and generalization capabilities of the MATRS algorithm, we constructed two scenarios of increasing complexity, building upon the 200 m × 200 m benchmark scenario. By expanding the collection area and increasing the number of sensor nodes and UAVs, we comprehensively assess the algorithm’s performance under greater challenges. The specific configurations for the three scenarios are summarized in Table 5.

**Figure 7:**
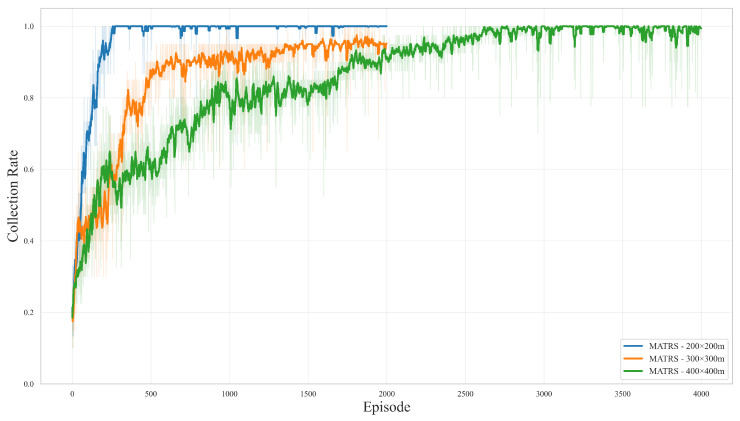
Learning curves of the MATRS algorithm across three different problem scales. Solid lines represent the moving average of the data collection rate, which smooths the learning trend. The faint, shaded area in the background of each curve illustrates the raw, unaveraged collection rate from each episode, showing per-episode performance volatility.

Figure 7 illustrates the data collection rate of MATRS as a function of training episodes across these three scenarios, providing a direct visualization of the relationship between convergence and problem complexity. In the benchmark scenario, the algorithm demonstrates exceptional learning efficiency, reaching and sustaining a 100% data collection rate after only approximately 250 episodes. As the environment scales to the 300 m × 300 m medium-scale scenario, the convergence time extends, with the algorithm requiring nearly 2000 episodes to achieve the same level of performance. In the most complex 400 m × 400 m large-scale scenario among those tested, the number of episodes needed for convergence further increases to approximately 3000.

It is also observed that the variance and instability of the learning curves are more pronounced during the initial training phase of the more complex scenarios. This is an expected outcome, attributable to the inherent difficulty of exploration within a vastly expanded state–action space. Nevertheless, the fact that MATRS ultimately converges to an effective policy that achieves 100% data collection in all tested scenarios provides compelling evidence of the framework’s robustness and promising scalability when applied to multi-agent tasks of increasing complexity.

The following table and figure detail the task completion efficiency and the final planned trajectories of the learned policies in the three scaled scenarios.

Table 6 summarizes the optimal number of steps required for MATRS to complete the data collection task in each scenario. In all three cases, the steps taken are significantly lower than the maximum episode length, indicating that the algorithm learns highly efficient policies rather than relying on “deadline-driven” strategies. Notably, when scaling from the medium to the large scenario, despite a significant increase in both the area size and the number of sensors, the required steps only increased by a marginal 5.2%. This is a direct result of adding a fourth UAV, and it demonstrates that MATRS can effectively leverage the collaborative potential of additional agents to parallelize the task, thus avoiding a linear decrease in efficiency as the problem scales.

**Figure 8:**
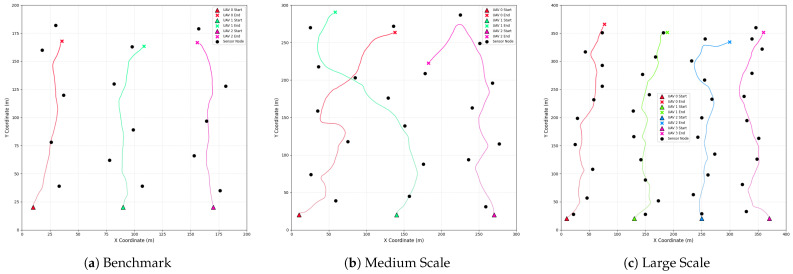
Visualization of the final learned trajectories by MATRS in the (**a**) benchmark, (**b**) medium-scale, and (**c**) large-scale scenarios. In each plot, the colored lines represent the trajectories of individual UAVs. For each UAV, a triangle marks its start point, and a cross marks its end point. Black circles denote the locations of sensor nodes. The axes are in meters (m).

The trajectory visualizations in Figure 8 intuitively reveal the sophisticated collaborative mechanism learned by the algorithm. In all scenarios, the UAV swarm exhibits a clear and efficient “task-space partitioning” strategy. Each UAV autonomously becomes responsible for a distinct, geographically contiguous cluster of sensor nodes. This results in an efficient, distributed coverage of the mission area that naturally avoids resource conflicts and redundant flight paths. The emergence of this intelligent cooperative behavior is key to the algorithm’s ability to solve large-scale problems efficiently and provides strong evidence for the scalability of MATRS in multi-agent path planning for data collection tasks.

#### 4.2.4. Scalability Limits and Practical Considerations

However, while this demonstrates effective scaling in our tested configurations, it is crucial to acknowledge the theoretical limits of this scalability. The self-attention mechanism at the core of our Transformer critic has a computational and memory complexity of $O(K^{2})$, where K is the number of agents. This quadratic scaling might become a bottleneck in scenarios with a larger number of UAVs, such as K > 20. Future work could explore modifying the UAVs’ communication topology or adopting a hierarchical approach to divide agents into sub-swarms, thereby achieving more efficient intra-group coordination.

Furthermore, bridging the gap between simulation and reality requires addressing key practical constraints. A paramount concern is the limited onboard energy of UAVs, which dictates their operational range and endurance. To this end, future work should focus on incorporating explicit energy consumption costs into the reward function or as a constraint to learn more energy-aware policies. Simultaneously, while our penalty-based approach encourages safe behavior, deploying UAVs in real-world environments necessitates stronger safety assurances. Integrating formal methods, such as control barrier functions (CBFs) or runtime safety shields, to provide hard safety guarantees represents another critical step toward making the MATRS framework field-ready.

## 5. Conclusions

In this paper, we addressed the complex challenge of cooperative path planning for multiple UAVs in agricultural data collection tasks, proposing a novel multi-agent reinforcement learning algorithm named MATRS. Our approach is built upon the Centralized Training with Decentralized Execution paradigm and integrates a SAC framework to ensure efficient exploration and policy stability. The core innovation of MATRS lies in its centralized critic architecture, which replaces conventional MLP networks with a Transformer-based encoder. This design leverages the self-attention mechanism to effectively model the complex inter-agent dependencies and accurately evaluate the joint action–value function, which is critical to learning sophisticated cooperative strategies.

Through a series of comprehensive simulation experiments, we have demonstrated the significant performance benefits and promising scalability of MATRS. Compared with established baseline algorithms such as MADDPG, MATD3, and MASAC, MATRS consistently exhibited faster convergence and greater stability across various data load scenarios. This superior efficiency is starkly highlighted when comparing MATRS with MASAC, where across all five data load scenarios, MATRS reduced the steps required for task completion by 10% to 30%. Furthermore, the visualization of the final trajectories provided compelling evidence of an emergent and efficient “task-space partitioning” strategy, where the UAV swarm autonomously divides the mission area for conflict-free coverage. This intelligent cooperative behavior, enabled by the powerful representation capacity of the Transformer critic, underscores the algorithm’s ability to solve complex coordination problems. Finally, scalability tests in larger and more complex environments confirmed the robustness and promising generalization capabilities of the proposed framework.

However, we acknowledge the limitations of this study. Our validation is currently confined to simulated environments, and the framework was tested using a homogeneous swarm of UAVs. These limitations motivate our primary directions for future work. In future work, we plan to validate the proposed algorithm in real-world scenarios to further assess its practical effectiveness and robustness. In addition, we will extend the MATRS framework to applications involving heterogeneous agents, where coordination among UAVs with different capabilities poses new challenges. Moreover, we plan to enhance the framework by incorporating more realistic constraints, such as ensuring Quality of Service (QoS), optimizing for Age of Information, and designing intelligent UAV recharging strategies.

The capabilities demonstrated by MATRS hold significant practical implications for the advancement of precision agriculture. The efficient coverage strategies are applicable to automating crop surveillance, where a swarm of UAVs can autonomously gather data to identify early indicators of crop distress—such as disease, water deficits, or nutrient imbalances—across vast agricultural expanses. In the context of irrigation management, the MATRS framework can empower a UAV fleet to perform rapid and systematic soil moisture mapping, enabling data-driven water resource management that optimizes usage and enhances crop yields. By automating and optimizing these critical data acquisition tasks, our framework serves as a foundational component for developing more scalable, responsive, and intelligent agricultural management systems.

## Figures and Tables

**Table 1:** Architectural and parametric comparison of MASAC and MATRS.

Aspect	MASAC (Baseline)	MATRS (Ours)
*Actor Network (Identical for Both)*		
**Input**	Local Observation	Local Observation
	Input Dim: $o_{\mathrm{local}}=50$	Input Dim: $o_{\mathrm{local}}=50$
**Hidden Layers**	2-Layer MLP ($d_{\mathrm{mlp}}=512$)	2-Layer MLP ($d_{\mathrm{mlp}}=512$)
**Sample Output**	Sampled Action from Gaussian Dist.	Sampled Action from Gaussian Dist.
	Output Dim: $a_{\mathrm{local}}=2$	Output Dim: $a_{\mathrm{local}}=2$
**Parameter Count**	≈291k	≈291k
*Centralized Critic Network (Key Difference)*		
**Input**	Flattened Vector of Joint Obs-Action	Sequence of Per-Agent Obs-Action Pairs
	Input Dim: $K(o_{\mathrm{local}}+a_{\mathrm{local}})=156$	Input Dim: $\left[K,o_{\mathrm{local}}+a_{\mathrm{local}}\right]=\left[3,52\right]$
**Hidden Layers**	2-Layer MLP ($d_{\mathrm{mlp}}=512$)	**Transformer Encoder**
		(2 Layers, 4 Heads, $d_{\mathrm{model}}=512$)
		+ **MLP Head** ($(K\times d_{\mathrm{model}})\to 256$)
**Output**	Single Joint Q-Value	Single Joint Q-Value
**Parameter Count**	≈344k	≈**6.73M**

**Table 2:** Key simulation parameters for the benchmark scenario.

Parameter	Value
Communication Bandwidth (*B*)	1 MHz
UAV Flight Altitude (*H*)	5 m
Channel Noise Power Spectrum ($\sigma^{2}$)	−110 dBm
Sensor Data Volume ($D_{\mathrm{node}}$)	10 MB (for benchmark)
Sensor Transmit Power ($P_{t}$)	20 dBm
Signal Reception Power Threshold	−60 dBm

**Table 3:** Key hyperparameters for reinforcement learning training.

Hyperparameter	Value
Discount Factor (γ)	0.99
Soft Update Rate (τ)	0.005
Episode Length ($T_{\max}$)	500
Total Episodes ($M_{\mathrm{episodes}}$)	2000
Batch Size	256
Replay Buffer Size	1,000,000
Policy Update Frequency	2
Actor Learning Rate	$1\times 10^{-4}$
Critic Learning Rate	$1\times 10^{-4}$
Temperature (α) Learning Rate	$1\times 10^{-4}$
Embedding Dimension	512
Number of Attention Heads	4
Number of Transformer Encoder Layers	2
Initial Temperature (α)	1.0
Target Entropy (ℋ)	−dim(𝒜)

**Table 4:** Minimum steps required to achieve 100% data collection under varying data loads. The results of the proposed MATRS algorithm are marked in bold. A dash (–) indicates that the algorithm failed to complete the task in that scenario.

Algorithm	10 MB	20 MB	30 MB	40 MB	50 MB
MADDPG	317	373	333	340	356
MATD3	–	349	387	–	464
MASAC	428	404	400	376	490
MATRS	**309**	**317**	**322**	**337**	**343**

**Table 5:** Configuration of the three scenarios for scalability testing.

Parameter	Benchmark	Medium Scale	Large Scale
Area Size	200 m × 200 m	300 m × 300 m	400 m × 400 m
Number of Sensors	15	20	40
Number of UAVs	3	3	4
Max Episode Length	500	800	1200
Total Episodes	2000	2000	4000

**Table 6:** Task completion performance of the final MATRS policy in the three scaled scenarios.

Scenario	Agents (Sensors/UAVs)	Converged Episode	Minimum Steps
Benchmark	15/3	∼250	309
Medium Scale	20/3	∼2000	709
Large Scale	40/4	∼3000	746

## Data Availability

The data presented in this study are available on request from the corresponding author.

## Appendix A

This appendix presents the cumulative reward curves for the data load scalability experiments discussed in Section 4.2.1. These plots illustrate the episode-by-episode performance of each evaluated algorithm, providing a detailed view of their learning stability and variance. For each curve, the solid line represents the smoothed reward trend, while the shaded area shows the range of the original, unsmoothed data.

A strong positive correlation is observed between the cumulative reward curves (Figure A1) and the data collection rate curves (Figure 5). This correlation validates that the designed reward model effectively guides the agents toward discovering the optimal data collection policy.
